# Stakeholder views on the acceptability of human infection studies in Malawi

**DOI:** 10.1186/s12910-020-0454-y

**Published:** 2020-02-05

**Authors:** Blessings M. Kapumba, Kondwani Jambo, Jamie Rylance, Markus Gmeiner, Rodrick Sambakunsi, Michael Parker, Stephen B. Gordon, Kate Gooding

**Affiliations:** 1 Malawi-Liverpool Wellcome Trust Clinical Research Programme, P.O. Box 30096, Chichiri, Blantyre, 3 Malawi; 2 Liverpool School of Tropical Medicine, Liverpool, UK; 3 Wellcome Centre for Ethics and Humanities and Ethox Centre, University of Oxford, Oxford, UK

**Keywords:** Human infection studies, Pneumococcal, Malawi, Acceptability, Ethics

## Abstract

**Background:**

Human infection studies (HIS) are valuable in vaccine development. Deliberate infection, however, creates challenging questions, particularly in low and middle-income countries (LMICs) where HIS are new and ethical challenges may be heightened. Consultation with stakeholders is needed to support contextually appropriate and acceptable study design. We examined stakeholder perceptions about the acceptability and ethics of HIS in Malawi, to inform decisions about planned pneumococcal challenge research and wider understanding of HIS ethics in LMICs.

**Methods:**

We conducted 6 deliberative focus groups and 15 follow-up interviews with research staff, medical students, and community representatives from rural and urban Blantyre. We also conducted 5 key informant interviews with clinicians, ethics committee members, and district health government officials.

**Results:**

Stakeholders perceived HIS research to have potential population health benefits, but they also had concerns, particularly related to the safety of volunteers and negative community reactions. Acceptability depended on a range of conditions related to procedures for voluntary and informed consent, inclusion criteria, medical care or support, compensation, regulation, and robust community engagement. These conditions largely mirror those in existing guidelines for HIS and biomedical research in LMICs. Stakeholder perceptions pointed to potential tensions, for example, balancing equity, safety, and relevance in inclusion criteria.

**Conclusions:**

Our findings suggest HIS research could be acceptable in Malawi, provided certain conditions are in place. Ongoing assessment of participant experiences and stakeholder perceptions will be required to strengthen HIS research during development and roll-out.

## Background

Human Infection Studies (HIS), sometimes referred to as human challenge studies, involve deliberately infecting healthy adult volunteers with a microbial pathogen, such as malaria parasites or typhoid bacteria. HIS are often used for vaccine testing, with volunteers vaccinated before infection to test potential vaccine candidates. HIS can be conducted much more rapidly and with far smaller sample populations than standard efficacy trials, which means HIS can accelerate vaccine development [1, 2]. HIS science has contributed to new vaccines in several areas, including typhoid and malaria [2–4]. While HIS have been conducted for decades in High-Income Countries (HICs), they are much newer in Low and Middle-Income Countries (LMICs) [2, 3]. The highest burdens of vaccine-preventable disease are found in LMICs, and different biological and environmental conditions, including population genetics and pathogen strains, mean findings from vaccine trials in high-income settings do not always apply [5]. Consequently, conducting HIS in LMICs holds the potential to help develop effective vaccines for these populations.

The deliberate infection involved in HIS science creates challenging questions for ethical practice and community engagement [6, 7]. Challenges are particularly apparent in LMICs. For example, informed consent and fair compensation are complex in contexts of limited research experience and high poverty; health systems may lack adequate facilities to monitor and treat adverse events, and regulatory guidelines may be limited [2, 4, 8–10]. The growing literature on HIS ethics suggests a range of ethical principles, including a strongly justified research question that can only be answered through HIS, independent review, qualified and experienced researchers, rigorous informed consent, safe selection of participants, minimisation of risks and no irreversible harm, protection of contacts and the environment, compensation that avoids undue influence, compensation for harm, and public involvement [4, 6, 8]. However, further evidence is needed to guide researchers and ethics committees on appropriate frameworks for HIS, particularly in LMICs [10, 11].

Stakeholder consultation to generate evidence on effective and acceptable approaches can help to inform ethical guidance and immediate HIS study design [2, 8, 12]. Such consultation can provide insight for critical decisions such as inclusion criteria, recruitment, informed consent, and comprehension testing, residential stays, compensation, withdrawals, and community engagement [4, 13].

### Undertaking HIS in Malawi

The Malawi-Liverpool-Wellcome Trust Clinical Research Programme (MLW) is planning *Streptococcus pneumoniae* HIS in Malawi (the Malawi Accelerated Research in Vaccines using Experimental and Laboratory Systems project, or (MARVELS)) [14]. This project builds on over a decade of pneumococcal HIS research at the Liverpool School of Tropical Medicine (LSTM), UK, and is predicated on the need for new pneumococcal vaccine in Malawi as current vaccines do not create herd immunity [15]. *Streptococcus pneumoniae (pneumococcus)* is a bacterial pathogen commonly found carried in the nose. If the pneumococcus spreads to the lungs, blood, or brain, it causes serious infection, including pneumococcal pneumonia, sepsis, and meningitis, respectively [16, 17]. In Malawi, pneumococcus is found in 90% of all children’s noses and 40% of adults with Human Immunodeficiency Virus (HIV) (10–20% adults without HIV) [15, 18, 19]. The project conceives a series of HIS, starting by establishing the feasibility of carriage in 12–36 participants. If effective, the project would move to further stages of vaccine testing, scaling up to around 150–400 participants per study depending on the stage and study focus. Volunteers would be exposed to pneumococcus through a saline solution dropped into the nose, and closely monitored for safety. Given uncertainties around participant contact and hospital access, a three-day residential stay is planned for volunteers following inoculation. The provided accommodation to participants is located in close proximity to one of Blantyre’s large private hospitals.

To reflect on the implications of undertaking HIS in Malawi, MLW and partners organised a workshop on the potential and challenges of HIS in Malawi in 2017. This workshop brought together government stakeholders, ethics committee members, and researchers, including people with experience of conducting and regulating HIS elsewhere in Africa and in HIC [2]. The workshop identified a need for community consultation to understand the acceptability and ethics of HIS in the Malawi context. This study responds to this recommendation.

### Aims of this study

Our immediate aim in conducting the research reported in this paper was to generate evidence on stakeholder perceptions and preferences that could inform MLW’s decisions about whether to proceed with HIS in Malawi and, if so, on MARVELS design and implementation. While the MARVELS team felt HIS had considerable potential for benefit in Malawi, a decision to apply for permission to conduct a feasibility study depended on knowing whether or not HIS would be welcome and socially appropriate. Approvals for pneumococcal HIS feasibility are still pending. The data presented in this paper were included in the ethics submission to support their review. We also aimed to support the wider development of ethical frameworks for HIS science in Malawi and other LMICs through information on stakeholder perceptions of ethical challenges and potential approaches in this context.

In considering the acceptability of HIS, we were interested in a definition of acceptability that involves more than tolerance [20]. MLW’s aim is that communities and other stakeholders should feel highly positive about HIS research in Malawi, seeing HIS as relevant and ethical, rather than just allowing HIS to take place. We wanted to find out whether pneumococcal HIS would be seen as worthwhile and ethical and how HIS could be designed to ensure it met stakeholder standards, expectations, and the highest ethical standards.

## Methods

We used Focus Group Discussions (FGDs) and individual interviews to examine stakeholders’ perceptions and opinions on the acceptability and ethics of HIS research in Malawi.

We conducted 6 deliberative FGDs [21] with MLW frontline research staff, medical students, and three sets of community representatives: chiefs (local community leaders), religious leaders, and members of MLW’s Community Advisory Group (CAGs). Frontline research staff included research nurses and fieldworkers, and we focused on staff involved in vaccine and other clinical trials that involved some procedures that would be used in pneumococcal HIS (e.g., nasal samples, health screening, and in-patient stays). The CAGs comprise representatives of active community-based organisations located in rural or urban Blantyre. CAG members were elected by their communities, and their main role is to advise MLW on appropriate engagement with communities for research. The chiefs and religious leaders involved in this study had no previous official engagement with MLW but were aware of MLW’s existence. In addition, some of these community leaders had previously participated in research conducted by other research institutions.

To allow participants to reflect and share considered perspectives, we conducted Follow-Up Interview (Fup) after 2–3 weeks with 3 participants from each FGD to identify any change in views or further reflections. We selected participants for follow-up interviews based on the opinions contributed in FGDs and level of participation, focusing on those who had expressed particularly critical or cautious views that we wanted to explore further, those expressing their views most strongly, and participants who had spoken little during group discussions. This strategy proved useful for ensuring we did not misinterpret original strong opinions and gathered views from those less confident to speak in a group setting.

We also conducted 5 Key Informant Interviews (KII) with a combination of Research Ethics Committee (REC) members, senior clinicians at the hospital where pneumococcal HIS (MARVELS) would take place, and district health management officials. The sample of participants is shown in Table 1. In later FGDs and interviews, no new issues emerged, so we concluded that data saturation had been reached and ended data collection [22].

**Table 1:** Stakeholders for FGDs, follow up interviews and key informant interviews

Method	Stakeholder group	Number of participants
Focus group discussion	Frontline researcher staff (research nurses and fieldworkers)	9 (2 FGDs, one with 4 and one with 5 participants) (9 females, 1 male)
	Community Advisory Group (CAG) members (Blantyre urban)	10 (5 females)
	Chiefs (Blantyre rural)	10 (4 females)
	Religious leaders (Blantyre urban)	10 (1 female)
	Medical students	10 (4 females)
Post-FGD follow-up interviews with a sample of FGD participants	Frontline researcher staff	3 (All females)
	CAG members	3 (2 females)
	Chiefs	3 (2 females)
	Religious leaders	3 (1 female)
	Medical students	3 (1 female)
In-depth interview	Research ethics committee (REC) members	2
	Senior clinicians	2
	District health management officials	1
Total		69 (33 females)

### Data collection procedures

Interviews and focus groups used a semi-structured topic guide (Additional file 1) with open-ended questions about views on HIS, including perceived benefits and concerns, particular procedures (such as inclusion criteria, compensation, and safety measures), the types of HIS that would be acceptable in Malawi and the overall ethics of HIS. As HIS research had not yet been conducted in Malawi, we anticipated that few, if any, FGD and interview participants would have heard of HIS or be familiar with HIS procedures. To ensure participants could give views based on some understanding of HIS, we provided information on HIS during each FGD and interview and encouraged participants to ask questions if they wanted more information. This approach drew on ideas of deliberate discussion, used in empirical ethics research to uncover participants’ informed, considered, and collective views on a normative question [23]. Information on the general HIS approach was provided at the start, and more specific information about the potential MARVELS plan and pneumococcal HIS was provided later once participants had given initial views. In addition, further information was provided as needed in response to questions from participants or where we noticed misconceptions about procedures that would be involved (e.g., that children would be enrolled, that the residential stay would involve quarantine, or that HIS would help the development of treatment rather than vaccine).

We were conscious that the views expressed by stakeholders would be heavily influenced by the information we provided. We wanted to avoid describing HIS in a way that only emphasised benefits and discouraged any critical views, but we were also cautious about creating fear and rumours based on misunderstandings (e.g., if we did not discuss planned procedures for safety). To balance this, we sought to present information neutrally and to focus on procedures that would be involved in HIS and documented experience in relation to safety (e.g., plans for a residential stay, and the number of severe adverse events recorded in LSTM). The information provided was developed through discussion among the study team. To encourage openness and reduce courtesy bias, we highlighted that there are different views about whether HIS is acceptable, that there was uncertainty about appropriate procedures, and that the MLW team needed stakeholder feedback and guidance. We also emphasised that the social scientists primarily responsible for conducting the interviews and FGDs were not part of the main MARVELS team. One of the MARVELS scientists who had worked on HIS in LSTM (KJ or JR) was involved in part of 4 FGDs to explain the detailed MARVELS procedures and answer questions from participants. We were conscious that their involvement might increase courtesy bias but decided it was important to explain additional details for participants and found that participants appreciated the opportunity to ask questions directly of people who had been involved in HIS in the UK. For FGDs where these scientists were involved, we allowed time, after they had left for further discussion, in case participants, were more reluctant to share critical views with the HIS scientists present. Follow-up interviews provided a further opportunity for participants to share any views they may have been reluctant to express in front of those involved more directly in HIS. This combination of deliberative FGDs and follow-up interviews has been effective for similar ethical topics in similar contexts [24].

FGDs with community representatives were conducted at a neutral venue, while follow-up interviews were held at places chosen by participants (their homes or workplaces). For MLW staff and medical students, FGDs and follow-up interviews took place at the MLW office in Blantyre, as the MLW setting is more familiar for these groups and so less likely to discourage open discussion. All KIIs took place at participants’ workplaces.

### Data processing and analysis

All FGDs and interviews were audio-recorded and transcribed, and those conducted in Chichewa were translated into English.

The analysis was ongoing during fieldwork, using an iterative approach to identify emerging themes that could be clarified or explored further through later data collection. We conducted thematic coding in NVivo using broad deductively defined themes (such as views on inclusion criteria or perceived benefits of HIS) and inductively derived sub-themes (such as impacts on household members or population benefit). We also used framework matrices to compare perspectives between different stakeholders. Two researchers (KG and BK) worked together on coding to compare interpretations and agree on a coding framework.

### Ethical approval

The study received ethical approval from the Liverpool School of Tropical Medicine and Malawi College of Medicine Research Ethics Committee. We sought permission from Principal Investigators at MLW and the College of Medicine to speak to frontline research staff and medical students, respectively. All participants received written information sheets and verbal explanations and gave written consent.

## Results

In this section, we present our findings under three broad headings. First, we describe stakeholders’ views on the acceptability of pneumococcal HIS, including perceived benefits, concerns, and views about whether overall pneumococcal HIS might be acceptable and ethical in Malawi. Second, we report stakeholders’ suggestions on the requirements for pneumococcal HIS to be acceptable, including appropriate models of consent, a fair selection of research participants, and the availability of medical support. Third, we describe views on HIS for other diseases beyond pneumococcus, to consider the acceptability of HIS that require different procedures.

### Views on the acceptability of pneumococcal HIS research in Malawi

#### Perceived benefits of HIS

All stakeholder groups saw potential benefits of pneumococcal HIS research in Malawi, centred on the potential to improve population health. Within this, key issues included the high burden of pneumococcal disease and the value for a vaccine suited to the Malawi population.

In relation to pneumococcal disease, almost all stakeholders focused on pneumonia rather than other types of pneumococcal disease (only medical students mentioned sepsis). Stakeholders noted the high incidence and severity of pneumonia disease and suggested that developing a vaccine would protect people from ill-health.

“I think it is welcome because pneumonia takes a lot of lives […] a vaccine to prevent pneumonia is really needed.” (Religious Leader, FGD6)

A vaccine developed specifically for the Malawi context was considered valuable given differences in genetic background and environmental context.

“It’s indeed right to conduct the research […] the drugs we have here were developed in Europe and maybe because of differences in climate and our bodies; those drugs don’t work here” (Chief, FGD4)

“These studies have been done in a setting like the UK, but it’s different from here, isn’t it? Here there is a number of things such as genetic and environmental factors that may have a bearing on how people respond to pathogens” (Senior clinician, KII)

While many stakeholders mentioned these benefits, some raised questions about the potential value of HIS research. In particular, while community participants seemed confident that HIS would deliver solutions for population health, a few key informants expressed uncertainty about the likely impact of pneumococcal HIS, perhaps reflecting awareness of both the unpredictability of research and the need to prioritise among competing research agendas.

“I am just interested to know why you want to do a HIS study on pneumococcus now in Malawi. We know that yes, *Streptococcus pneumoniae* is a big problem, […], so what’s changed for this to come up now? […] what will be its impact?” (Senior clinician, KII)

#### Perceived concerns about HIS

While stakeholders saw HIS as having potential benefits, they also had concerns. Key issues emphasised by all stakeholder groups were safety and community reactions.

##### Participant safety

On safety, the deliberate infection was initially perceived as carrying significant risks by many stakeholders. However, when we explained the focus on pneumococcal carriage (that is, only observing ability of the bacteria to live in the nose rather than causing pneumonia or other pneumococcal diseases), the prevalence of carriage and that there is low rate of adverse events in similar HIS in the UK, many stakeholders were reassured about safety.

“If you are just monitoring whether someone has it in their nose without it necessarily affecting that person, then you aren’t causing any harm for people to be scared” (Religious leader, FGD6-Fup)

“I don’t think it will have any risks and I don't think it’s as intense as I thought before […] with how you have explained that you’re just looking at carriage, I think it’s safer” (Frontline research staff, FGD1-Fup)

Nevertheless, safety concerns remained among some stakeholders, particularly those involved in medical care and research. Key issues were the unpredictability of individual biological reactions to the pneumococcus and limited healthcare facilities to manage severe adverse events in Malawi.

“People react differently to those agents, maybe because of their difference in biology, so just in case someone experiences the infection, and dies, […] so as researchers, how prepared are you to handle such…?” (Medical student, FGD5-Fup)

“Obviously you would want to do it in a setting whereby if a participant is sick, you are able to give all the treatment that is necessary, such as High Dependency Unit care or Intensive Care Unit. But in Malawi it’s a bit tricky because we already struggle with resources to take care of patients that are very sick.” (Senior clinician, KII)

Even when participants felt procedures were largely safe, some were concerned about the psychological impact of participation in HIS related to fear of infection.

“That psychological part of knowing for sure I have been infected - I think they could not interact normally or be as productive as they could, because they would have that psychological mind-set that ‘I may be sick, let me restrict myself’.” (District Health Management, KII)

##### Negative community reactions

Partly linked to this concern about fear, all stakeholder groups expressed concern about negative community reactions to HIS, believing communities would see HIS research as high-risk and that misconceptions and rumours were likely. For example, participants mentioned the potential for rumours about the purpose of deliberate infection, links to witchcraft, and the intentions of non-Malawian researchers, particularly because MLW may be seen as a foreign institution.

This concern about community reactions partly reflected the context of recent rumours around ‘bloodsucking’ in Malawi: in 2017, there were widespread rumours about bloodsuckers, with related community violence and some disruption to community-based research [25, 26]. This recent experience heightened sensitivity to potential community distrust:

“There are concerns, people don’t trust their fellow humans. [or] believe that an organisation has come to help, for example, the issue of bloodsuckers. So to just enter a village without proper communication, I tell you - you can be chased away.” (Religious leader, FGD6)

Potential community distrust of HIS was seen by all stakeholder groups as likely to limit the recruitment of HIS volunteers.

“Recruiting participants could be a problem in most communities due to misunderstanding the information and not completely trusting the people who conduct the research.” (Religious leader, FGD6)

In addition, frontline researchers saw the potential for rumour as carrying risks for MLW’s reputation and for the safety of frontline staff in other studies.

“For those who go into the community, like the field workers, or even the hospital based research staff, their security and safety […] have you thought about the safety?” (Frontline research staff, FGD2)

“We are trying hard to build…we have established community trust, of which we can say that we are not 100% good; we have got some areas where we are also lacking, and we are trying hard to maintain that. So, bringing in HIS […] we might end up ruining the trust that people have” (Frontline research staff, FGD2)

#### Reflections on the overall acceptability of pneumococcal HIS

As illustrated in the previous sections, many participants described both benefits and concerns around pneumococcal HIS. To understand overall acceptability, participants were asked whether they thought HIS research should go ahead in Malawi. Most felt HIS would be acceptable provided researchers addressed certain important conditions (conditions we explain below). Views ranged from high levels of enthusiasm to more uncertainty but with willingness to consider that HIS could be appropriate.

“Had it been there were these studies done before, maybe I would have said ‘Yeah it’s OK, this can be done’. But…I am on the 50-50, yes or no because it’s the very first time and I am thinking ‘how are they going to handle issues of safety?’” (REC member, KII)

“As a Malawian citizen I believe this research is really necessary, because people in our country are struggling […] in our hospitals, doctors try to save lives but lives are being lost. Now researchers have suggested new methods that can be tried to save lives.” (CAG member, FGD3-Fup)

Positive overall views of HIS were often related to the awareness that many existing medicines were developed through research with a few people for later public use. Research was therefore considered a normal and necessary step to improving population health, and so acceptable even if it may involve risks and burdens for some individuals.

This same idea of benefit for the greater good was reflected in discussions explicitly about the ethics of HIS, and specifically whether HIS research would meet principles of justice and ensuring a ‘fair offer’ for participants, with risks and burdens of HIS adequately balanced by the benefits [27]. Many stakeholders emphasised that some risk or burden for participants was outweighed by the potential for population health benefit, or discussed the positive aim behind HIS:

“I feel it is a fair thing … you would get vaccines developed quickly from a small number of volunteers which can benefit the population […] so I feel like it has all those benefits that could outweigh the negative parts.” (District health management, KII)

Some staff, community leaders and medical students shared these sentiments.

However, views here varied, and a few stakeholders were concerned about lack of individual benefit:

“It sounds to me like the benefit is more for the study than the participants, I can’t see what they have to benefit, rather than contributing to whatever vaccine may be developed in the long term.” (Senior Clinician, KII)

To promote an adequate balance of benefits and burdens, participants identified a range of conditions and procedures needed to address concerns around safety and community reactions and ensure pneumococcal HIS was ethical and acceptable. These conditions are discussed below.

### Conditions required for the acceptability of pneumococcal HIS

As reported in the previous section, views on the ethical acceptability of pneumococcal HIS in Malawi were sophisticated and varied, suggesting that HIS might be acceptable under some conditions but not others. In this section, we report on stakeholders’ views on what they considered to be the requirements for ethical and acceptable pneumococcal HIS.

#### Voluntary and informed consent

All stakeholder groups highlighted voluntary and informed consent as a key condition affecting the acceptability of HIS. The priority placed on informed consent was evident in a discussion about whether a pneumococcal HIS as MARVELS was just and constituted a ‘fair offer’ for participants. Those stakeholders who viewed the MARVELS study as ethically acceptable often emphasised the informed, voluntary consent process as a basis for this judgement.

“That is justice because the participant will be informed; they have got the information and volunteered. If someone is volunteering, it means there is justice between you and them.” (Chief, FGD4-Fup)

“A good and thorough information I think will balance [the benefit and burden], where they will get the right information, and it has to be exactly what is going to happen.” (REC member, KII)

As these quotes show, discussion about informed consent emphasised both full understanding and voluntary decision making. Both aspects would be expected in most health research, but participants emphasised that explanations should be particularly clear and open with HIS, given concerns about community misconceptions, perceived potential for risk from deliberate infection, and complexity of information.

“They really need people who have got a strong understanding of things […] this time we are actually introducing a foreign body in the human body, right? So, this thing, it needs somebody to have a clear type of understanding.” (Frontline Research staff, FGD2)

While the overall emphasis was on transparency, a small number of participants raised concerns that the initial description of HIS procedures might cause alarm if handled badly. One CAG member, for example, suggested that people should not be told that HIS involved introducing bacteria as people would see this negatively, although other CAG members disagreed and emphasised the need for openness. One medical student expressed uncertainty about how to balance openness and avoiding concern, given that the idea of being infected may cause fear:

“We want the person to be informed, but then I think we need to be careful with these details because we may end up scaring people. […] I don’t know how we can put it because we need to find a balance between keeping them informed and then not scaring them away, because it’s really scary.” (Medical Student, FGD5-Fup)

Involvement of family members in consent was discussed by many stakeholders, reflecting a concern not to harm family relationships or household livelihoods and dominant norms around permission from family members, particularly for women. Again, enabling discussion with family is standard practice for consent in health research, but it was particularly emphasised for MARVELS because the planned three-day residential stay would take participants away from home

“People at home are supposed to agree with you so they are aware where the participant is and what is happening.” (CAG member, FGD3)

Scope for withdrawal was also an aspect of consent with more specific implications in HIS research. Some medical students and research staff raised questions about permission to withdraw, given the need to take antibiotics to clear any infection and saw this permission as important for voluntary consent.

“Maybe the person has volunteered himself, and then you have injected, you have started medications, maybe the medications are supposed to run maybe for the week. And then in the consent we have a part where it says the person can accept but at any time can withdraw. What if the medications haven’t yet finished, but the person maybe didn’t understand, and says I can’t continue, and you can’t say “no you have to, you have to!” What can happen in that situation?” (Frontline Research staff, FGD1)

Research staff felt safety and the right to withdraw could be balanced through sufficient explanation and ensuring participants fully understood planned procedures:

“We will explain to them that we will give you the bacteria and after that we will see if it can go by itself, but if it doesn’t and you have fever or whatever, we will be giving you some medication. So, if you withdraw, maybe if something can happen at home, there will be no problem. You can get this medication if you are not willing to continue.” (Frontline Research Staff, FGD1-Fup)

The perceived importance of informed and voluntary consent had implications for views about acceptable recruitment approaches. The MARVELS team was considering using flyers or adverts distributed through places such as college notice boards or social media, with phone numbers to contact for further information. Stakeholders welcomed this approach as avoiding pressure to take part (perhaps in contrast to the more typical of face to face recruitment in communities).

“I support the flyers, they are really good because when one reads it properly, they will be able to make a decision to say ‘aaah, I think I should take part in this research’, because they will read everything for themselves unlike just being told.” (CAG member, FGD3-Fup)

“I think it [use of flyers] will be of advantage, because people will come on their own will, we'll not coerce them to join the study.” (Frontline Research staff, FGD2-Fup)

Beyond specific procedures for recruitment and consent, the value placed on informed and voluntary participation also affected views on other procedures, particularly inclusion criteria and compensation, as discussed below.

#### Fair selection of participants

Inclusion and exclusion criteria for HIS participants were seen as an important consideration for acceptability among all stakeholder groups. Participants in focus groups and interviews were asked openly about criteria they saw as important and for their views on criteria being considered by the MARVELS team. Views on groups of who should be either targeted for participation or excluded reflected concerns related to informed and voluntary participation, community interest and misconceptions, equitable opportunities for participation between different population groups, burden on livelihoods or other activities, and ensuring research value and validity through a sample population considered relevant and unbiased - principles that were sometimes in tension.

The health status of HIS participants was raised by all stakeholder groups as an important consideration. Stakeholders recommended excluding people with long-term or acute conditions that might increase vulnerability to pneumococcal infection, as well as people allergic to the antibiotics used to clear the infection and those with other contraindications. They urged thorough screening, particularly because potential volunteers may be unaware of underlying conditions.

“I would be worried about […] people who have diseases that would lead them to be vulnerable to any sort of infection. So, people who have chronic comorbidities like a heart failure patient or somebody with chronic renal dysfunction.” (Senior clinician, KII)

“I think this research is suitable for someone who has been screened, someone who has no health problem, so that when they are infected, their body will easily fight the infection.” (CAG member, FGD3)

Views on inclusion of people with HIV were mixed, with concerns for safety but also for equity and relevance of the findings for a high-risk group. Many felt people with HIV should be excluded due to vulnerability to infections. However, some also discussed the need to ensure any resulting vaccine to be suitable for people with HIV, given their susceptibility to pneumococcal infection.

“This vaccine is not only going to those who are HIV negative, […], so don’t you think that we will also need to study those who are on ART, if we give them this vaccine, how is it going to work? Or like are we not leaving them aside? (Frontline research staff, FGD1)

As one way to balance these concerns for safety, equity and relevance, senior clinicians felt people with HIV might potentially be included if they were virally suppressed, though they felt more information was needed to make this decision:

“In my view, I don’t necessarily see that as a severe contraindication, if we have evidence that they have an undetectable viral load, but I don’t know” (Senior Clinician, KII)

Beyond criteria around health, stakeholders discussed three criteria under consideration by the MARVELS team: restricting participation to medical students, people fluent in English, and those with higher levels of education. The MARVELS team proposed these criteria to ensure potential participants could fully understand study information and to facilitate discussion with MARVELS clinicians, some of whom are British and without fluent Chichewa. Stakeholders saw potential benefits of these criteria for informed consent, and some supported restricting participation to these groups.

“I think the idea of involving medical students will be better because considering the…. understanding the concept, it’s easier for us (medical students), but for people out there it’s not going to be easy.” (Medical student, FGD5)

However, many stakeholders thought focusing only on these groups would be inequitable and deny opportunities for participation to others.

“If we say that participants should be determined by a certain level of education, we are being biased. Someone may be able to understand but not be educated to the standards you want.” (Chief, FGD4)

A further concern was that restricting participation might affect community views of the relevance of the research if participation was later expanded, or of the resulting vaccine.

“If you focus only on educated people, there will be a lot of questions around equity, whether the findings can be trusted, safety of volunteers and being unsure if the research has even started. It would be better to recruit some educated and some uneducated people, so the results represent both sides.” (CAG member, FGD3-Fup)

To address these concerns, some suggested broadening the sample, and involving Malawian clinicians in the research team or using translators to enable communication, as they had seen with other studies or in hospitals where translators assist English-speaking clinicians.

#### Adequate medical care

Medical support to ensure participant safety was viewed as critical for the acceptability of pneumococcal HIS research in Malawi. Key discussions related to the provision of adequate healthcare, the planned residential stay, and other measures designed to ensure safety.

##### Healthcare facilities

Stakeholders emphasised the need for high-quality healthcare facilities and procedures of the same standard as those in HIC:

“The standard should be better than the ones used in Liverpool, because in Liverpool obviously they have more resources for controlling […] let’s say things get out of hand, as compared to here, we do not have a lot of resources that can help us to control. So, monitoring will have to be of the best standard.” (Medical student, FGD5)

Participants also emphasised the importance of adequate laboratory facilities and experienced staff with the skills to support volunteers if they develop an infection. These technical and ‘backroom’ aspects were mentioned particularly by stakeholders from medical and research settings.

“It’s fine to do it but after we have made sure that everything is in place, we have a laboratory that is of high quality, the inoculum will be kept there safe, we have the expertise.” (Senior clinician, KII)

As well as staff with experience in HIS and pneumococcus, some participants mentioned the need to involve local staff for both, building research capacity and because of their existing trust by participants.

“You need to include local clinicians. Clinicians who are well known by people and who also should develop skills in carrying out that type of study.” (REC member, KII)

##### Residential stay

As previously described, the MARVELS team plans a three-night residential stay for participants following inoculation at an accommodation that is located in close proximity to one of Blantyre’s private hospitals. This residential stay was generally welcomed by stakeholders as important for safety, allowing easy access to medical care and the research team.

“It’s good you thought of arranging the residential stay to protect participants, because they will be close to Mwaiwathu hospital (private hospital in Blantyre). It will be safe for participants.” (Chief, FGD4)

However, while the residential stay was welcomed for medical support, there were some concerns about the impacts on household members left behind, and community participants in particular worried about the impact on livelihoods if the breadwinner is away from home. Some participants also mentioned that people may dislike being away from home or feel trapped in the hostel.

Views on an alternative non-residential option were mixed. Some felt this could not be considered for initial HIS studies, but others felt a non-residential option should be allowed, particularly if volunteers live close to the hospital or have good transport and communication access in case of adverse events.

“I think if the person stays in the city or has a car, it won’t be difficult; if they face any problem, they can rush to the hospital straight away.” (CAG member, FGD3-Fup)

##### Additional safety measures

Several additional measures were proposed by the MARVELS team as part of a package of medical support, largely following procedures used with pneumococcus HIS in the UK. This included providing an emergency package of antibiotics and a thermometer so that participants could take their temperature daily to monitor reactions to inoculation and treat themselves in case they could not access medical care in an emergency. Stakeholders generally saw this package as useful. However, many raised concerns about limited understanding of thermometer use and improper use of antibiotics, particularly among volunteers with less education or no medical training.

“If it’s the medical students then it’s OK, but if it’s not, it’s better they just come to be checked […] if he is not that well educated, if they just feel a fever, you are not sure that they will actually take the drugs as they have been advised…some may even overdose.” (Frontline Research staff, FGD2-Fup)

“Our friends in the UK are more advanced; they know how to use a thermometer, whereas someone from Malawi doesn’t know how it’s properly used.” (CAG member, FGD3-Fup)

To address this potential for misunderstanding, stakeholders suggested clear explanations for HIS volunteers about how and when to take the drugs, or follow-up visits at home by the research team to monitor antibiotic use.

Other medical support procedures proposed by the MARVELS team were generally seen as adequate, including 24-h access to a study doctor, availability of a research team member at the residential hostel, a sequence of monitoring visits to check for symptoms, and daily phone contact by the study doctor. With these procedures in place, most stakeholders felt reassured about safety.

“There is protection. If they see that a participant’s health has changed, they will rush them to the hospital, showing that there is help. So, we cannot have any concerns.” (Chief, FGD4)

#### Compensation and reimbursement

Compensation of volunteers participating in HIS research was identified by all stakeholder groups as important for acceptability. Within this, many participants mentioned reimbursing HIS volunteers for transport costs (for example, to attend monitoring check-ups at the hospital), and compensation for time away from income-generating activity. The latter was particularly important given the three-day residential stay.

“If he earns 2,000 a day [about £2], then that 2,000, you need to cover it for those days he participates in your study” (CAG member, FGD3-Fup)

Stakeholders also discussed compensation for risk. Some felt compensation for HIS participation would be higher than for other studies as they perceived HIS as higher risk:

“For the HIS I am sure we would go for a bit higher looking at the risks associated.” (REC member, KII)

However, others thought compensation should be similar to other studies, to avoid reducing recruitment for other studies or creating undue inducement. Concerns about undue inducement were mentioned by several stakeholders and considered a particular risk for less educated or lower-income groups.

“Here in Malawi, we have a lot of people who are poor, and poverty might be one reason they join the study, because of the incentives. They completely don’t understand the study, but because they want to make ends meet, they’ll just join.” (Medical student, FGD5)

Views on the amount of reimbursement and compensation varied widely, with suggested figures ranging from 15,000 [about £15] to 300,000 [about £300] for the 3-day residential stay, plus additional amounts covering transport for hospital visits. Often stakeholders found it hard to indicate specific amounts and suggested that compensation be determined by researchers based on standard practice and guidance.

“I feel like every organisation has its way of providing incentives and it would also be best to look [at] what do other organisations recommend on the type of incentives you can give to someone when you are involving his life and his health.” (Medical Student, FGD5)

In relation to how reimbursement and compensation should be given to volunteers, most stakeholders felt it should be spaced throughout the study, to cover costs as they arise, and so reduce burden, and to support retention.

As well as compensation for time and burden, stakeholders indicated the importance of insurance and compensation in the event of severe adverse events.

“As long as there is an agreement that you will take care of everything if the volunteers fall sick or die while participating, then we don’t have concerns.” (Chief, FGD4-Fup)

#### Regulation

Stakeholders emphasised the need for careful review and approval of any HIS in Malawi by research ethics committees (RECs) and other regulatory bodies such as pharmacy, medicine, and poisons body (PMPB), to protect the safety of participants:

“They have to give approvals to show that they have met the standards, and they are not bringing any kind of harm to these people.” (Medical student, FGD-Fup)

However, participants involved in medical care and research expressed concerns over the lack of standard regulatory guidelines on HIS to guide the local regulatory bodies.

“But also making sure that there are clear guidelines from the regulators […] because this will be the first study. I don’t know if our regulators have got guidelines to conduct this type of research” (Senior Clinician, KII)

#### Community engagement

Community engagement was considered by all stakeholders as a key component if HIS research is to be accepted in Malawi. As previously described, the potential for negative community reactions and distrust in HIS was a key concern for all stakeholder groups. However, many emphasised that adequate community engagement to promote understanding could overcome this concern:

“There should be enough awareness raising among everyone, so they know the importance of HIS. This will ensure they understand what will happen in the study and they will be able to explain to other people who might be creating stories about it.” (Religious leader, FGD6)

Comments on community engagement focused on raising awareness, sharing information about HIS, and providing an opportunity for people to ask questions. However, a few stakeholders also mentioned more two-way engagement with community input to HIS design, particularly seeking feedback from participants once HIS research begins.

On approaches for community engagement, participants suggested a range of channels, including working with chiefs, community opinion leaders, and the media to reach potential participants and wider communities.

“I think the channels to use are chiefs, CAG members, or churches. […] even radio and TV stations so that many people hear the information.” (CAG member, FGD3-Fup)

Community stakeholders also emphasised the presence of a HIS scientist during any community engagement activity to respond to any questions and give clear information.

“You may face challenges with lots of questions. As we (chiefs) are holding meetings, you [the scientist] should be present to explain things to people clearly.” (Chief, FGD)

### The acceptability of other types of HIS beyond pneumococcal studies

The views above relate to HIS focused on pneumococcal carriage. Participants were also asked for views on the acceptability of HIS for other diseases that require different procedures. Stakeholders had mixed views about the acceptability of other types of HIS, particularly HIS, such as malaria or typhoid, that are more likely to cause symptoms or require a longer residential stay or quarantine.

The higher likelihood of developing symptoms was seen by some stakeholders as a concern for safety, although others felt it could be justified by the high burden of the disease considered (for example, malaria) and acceptable with adequate medical support, including close monitoring.

“I think people will be more scared of the higher risks. But still more it’s worth doing it because like I said about malaria, there are a lot of people who are affected by malaria” (Medical student, FGD-Fup)

A longer residential stay or quarantine was considered positive for safety (including protecting participants and stopping the spread of infection to the public), but stakeholders mentioned potential negative impacts on family and livelihoods and concerns around participant comfort. Reasonable compensation and clear information on the procedures were identified as preconditions for acceptability.

“Let’s say the man is a breadwinner at home – staying for 20 days is difficult. […]. In that situation, you will provide not only transport but consider an amount to match the income he is missing.” (CAG member, FGD-Fup)

Several stakeholders suggested starting with pneumococcus and then gradually expanding to other, potentially more challenging, diseases if initial HIS go smoothly and produce useful findings.

“Because this is just the first HIS research starting in Malawi, it might be a bit risky in general; but I think as we go along we will see that maybe it’s going on well with the experience and there will be no problems with other types.” (Frontline research staff, FGD-Fup).

## Discussion

Our findings suggest that pneumococcal HIS research could be acceptable in Malawi, provided certain conditions are met. Many stakeholders saw HIS as potentially beneficial for population health, but they also had concerns, particularly related to safety and community reactions. Acceptability depended on approaches to aspects such as inclusion criteria, compensation, medical support, and community engagement. Many of the conditions highlighted by stakeholders reflect those in existing guidance on HIS, and in ethical guidance on biomedical research in general in LMIC. In this section, we compare our findings to existing guidance and draw out implications.

Many of the conditions for the ethical conduct of HIS proposed in existing guidance were discussed by stakeholders in Malawi. For example, stakeholders mentioned achieving an adequate level of informed consent, fair selection of volunteers, appropriate compensation, safety measures to minimise potential harms and discomforts, and robust community engagement [2, 4, 28, 29]. Stakeholder views helped to indicate what these principles might mean in the Malawian context.

Informed and voluntary consent was seen by many participants as a central condition, and critical for ensuring HIS is ethical and a fair offer. The idea of providing information through flyers or websites (rather than face to face) was considered potentially valuable for supporting voluntary decisions but needed to be accompanied by opportunities for an in-person discussion. This approach is in line with suggestions that recruitment for HIS participants should involve the distribution of advertisements on flyers and posters, followed by a comprehensive information package and information meeting [29]. Using flyers as a first step in the information chain could potentially miss people who are illiterate but who would be interested in taking part. Experience with other research in Malawi suggests that information about opportunities to participate in HIS would spread by word of mouth, so it may reach people unable to read flyers [20]. Inclusive forms of communication that reach all potential participants should be considered for future HIS, for example, ensuring providing engagement material in local languages.

Importantly for study design, ensuring informed and voluntary consent depends on more than the recruitment process, and stakeholders discussed the impacts on the consent of inclusion criteria and compensation.

In relation to inclusion criteria, stakeholder views reflected principles and potential tensions indicated in existing guidance. In particular, existing guidance notes the need to promote safety by potentially limiting participation to those with English or more education, but also the potential inequity of these exclusions [5, 30]. The compromise suggested by some participants in our research was to start with a narrower group of participants and potentially broaden inclusion criteria as experience with HIS grows. However, views varied, with some stakeholders prioritising safety and others equity (primarily community members, who might be excluded). Another concern highlighted by our participants in relation to limiting HIS to more educated groups was the need to focus on groups most affected by the target disease. This may partly reflect limited explanation during FGDs about the use of follow up on trials after HIS that test vaccines in a broader population. Further public engagement that explains the sequencing and discussed the extent to which relevant results can be achieved in non-target groups would help to consider and assess this concern.

A related angle not discussed in the HIS literature but of concern for some stakeholders in Malawi was the need for communities to perceive the study as relevant to them, not just for the results to be medically applicable; limiting inclusion was seen as potentially limiting wider community interest and support, and potentially future vaccine uptake.

The restriction to healthy adults proposed in existing guidance [4, 29] was widely supported by stakeholders in Malawi, along with the need for careful screening [2, 31]. The need for screening held a particular significance in the Malawian context where HIV rates are high, and status is often unknown [32]. Views on inclusion of people with HIV reflected the same tensions between safety, equity, and relevance seen with restrictions based on education and medical training: stakeholders recognised the need for people with HIV to have access to a vaccine that works for them, but also had concerns about adequate immunity.

Views on compensation also reflected some of the principles and tensions in the literature [2, 7, 9, 33]. Some stakeholders felt HIS volunteers should receive higher amounts, in line with suggestions in some guidance [34]. However, other stakeholders were concerned about undue influence, a concern widely shared in literature [2, 13, 28] and of particular significance in LMIC contexts where poverty may increase the value of study compensation [10]. Existing research in Kenya shows compensation can be a key driver of participation [10], but other studies in Kenya suggest compensation did not involve undue influence, and also did not set a precedent – another concern for some stakeholders in Malawi [8]. While many stakeholders suggested amounts for financial remuneration, some talked about access to health care as a potential form of compensation, reflecting the costs of accessing high quality (private sector) health care in Malawi.

Stakeholder discussions of compensation often highlighted the impact of participation on livelihoods and households, reflecting a context where many people have minimal if any savings, no formal employment, and rely on daily earnings through small-trading or other self-employment. In this context, even a short break in work can have significant effects on household livelihoods. Restricting participation to more educated groups may mean participants are more likely to be in formal employment, such that this is less of a concern. However, impacts on household well-being should be discussed with potential participants during recruitment and assessed through future study monitoring.

Many stakeholders suggested that compensation levels should be decided by the research community. In line with this, compensation levels for MARVELS will be discussed with the REC and based on recent guidelines for Malawi [35, 36],^1^ but ongoing monitoring to understand participant motivation will help to assess whether amounts and forms of compensation are appropriate.

The need for medical support to minimise risks and protect participants from harm was widely discussed, reflecting concern for safety. Availability of adequate medical facilities in case of harm was a concern for participants, given the constrained resources of the health sector in Malawi and consequent impacts on access to timely and quality care. Participants were generally reassured by plans for regular monitoring of participant health, continuous access to clinicians, and the residential stay to facilitate quick hospital access; an approach used effectively for HIS in similar contexts [4].

Community engagement is noted in some existing guidance on HIS but received heavy emphasis from stakeholders in Malawi. This emphasis on working with communities is seen in wider guidance on biomedical research in LMIC [2, 5, 10]. Stakeholders expected critical public reactions, at least initially, reflecting previous experience of public unrest and rumours affecting research in this context. Stakeholder views tended to emphasise providing explanations and informing the public, with an instrumental approach of using engagement to support the effective conduct of research. While sharing information will be part of public engagement, ongoing two-way dialogue is needed, as started through this consultation. This two-way approach is core to guidance on public engagement in LMICs [37], and needed both to inform the acceptable design of future HIS and for the intrinsic value and ethical importance of public involvement.

A key condition highlighted in existing guidance that any HIS study must have a strong rationale that can only be achieved through HIS, received less critical attention from our participants. A concern for the importance of research was reflected in numerous comments about the value of research on pneumonia. However, the need to use HIS rather than other study models was not questioned. Many benefits of pneumococcal HIS identified by stakeholders – such as addressing the high burden of pneumonia and improving vaccines – are not necessarily specific to HIS research and might potentially be achieved through other types of pneumococcal research. This assumption that HIS was required may have reflected trust in the expertise of researchers and a lack of familiarity with different types of vaccine research among many stakeholders, combined with a description of the rationale for HIS within FGDs and limited explanation of alternative approaches. Similarly, it was assumed by almost all participants that HIS would lead to effective vaccines, with only one senior clinician pointing to potential uncertainty in study impacts. HIS are one step in a series of studies needed to bring new vaccines to the market, but HIS researchers need to maximise the potential for impact through focusing only on questions with public health importance [2, 13], and through engaging with academic, pharmaceutical and policy stakeholders to advance translation of findings and progress along the vaccine development pipeline. This includes promoting access to any future vaccines among research communities, in line with broader ethical obligations regarding post-trial access in LMICs [38]. Providing this clarity would be in line with proposals that HIS should have a publicly available rationale that includes explaining why HIS is needed rather than alternatives [4].

Our assessment of acceptability is preliminary and subject to limitations. Participants were unfamiliar with HIS, and their views will have reflected the information provided during focus groups and interviews. While we sought to ensure information was adequate and neutral, the provision of alternative information or research conducted by people independent of MLW may have resulted in different findings. In addition, views are at this stage hypothetical, and perceptions may change when HIS starts. We plan to continue embedding social science in MARVELS and future HIS in Malawi to understand participant experiences and views of people who choose not to take part, to develop our understanding of acceptability, enable ongoing adaptation of HIS study design to optimise acceptability and to inform discussions on ethical approaches to HIS in LMIC settings.

## Conclusion

This study was the first investigation of HIS research acceptability in Malawi. Our findings highlight a range of concerns and issues to consider in the design of HIS. Within MLW, the findings informed the decision to proceed with a pneumococcal HIS feasibility study and were used in the study design. Summarised findings were also shared with the Malawi national ethics committee to support their ethical review of the feasibility study, and with the MARVELS funder. The findings highlight the importance of community and stakeholder engagement, both during initial consideration and design of HIS, and an ongoing dialogue to build mutual understanding and ensure any HIS research is acceptable.

## Supplementary information

**Additional file 1.** Topic guides for focus groups and stakeholder interviews. As described in the methods section, we have included the topic guide that we used to conduct focus group discussions, stakeholder interviews and follow-up interviews.These topic guides were drafted at the outset of the study but used flexibly to respond to issues raised by participants and adapted and refined during the fieldwork in response to emerging findings and reflections on previous interviews.

## Data Availability

The datasets generated and analysed during the current study are not publicly available due to them containing information that could compromise research participant privacy/consent, and because participants did not give consent to share the data beyond the research team.

## Abbreviations

CAG: Community Advisory Group
FGD: Focus Group Discussion
Fup: Follow-Up Interview
HICs: High-Income Countries
HIS: Human Infection Studies
HIV: Human Immunodeficiency Virus
KII: Key Informant Interview
LMIC: Low and Middle-Income Countries
LSTM: Liverpool School of Tropical Medicine
MARVELS: Malawi Accelerated Research in Vaccines using Experimental and Laboratory Systems project
MLW: Malawi-Liverpool-Wellcome Trust Clinical Research Programme
NCST: National Commission for Science and Technology
REC: Research Ethics Committee

## Footnotes

1 The National Commission for Science and Technology (NCST) (2019) provided guidelines on compensation suggest that: “The level of compensation for research participants be determined on a case-by-case basis and dependent on the nature and design of a study” [36]. Gordon et al. presented remuneration tables to guide researchers and regulators on ways to calculate compensation based on study time and burden [35].
