# Supplementary material for: Stakeholder views on the acceptability of human infection studies in Malawi
Source: BMC Med Ethics. 2020 Feb 5;21:14. doi: 10.1186/s12910-020-0454-y (PMC7003337; doi:10.1186/s12910-020-0454-y)
Supplement: Supplementary file 1 — Additional file 1. Topic guides for focus groups and stakeholder interviews. As described in the methods section, we have included the topic guide that we used to conduct focus group discussions, stakeholder interviews and follow-up interviews.These topic guides were drafted at the outset of the study but used flexibly to respond to issues raised by participants and adapted and refined during the fieldwork in response to emerging findings and reflections on previous interviews. [file 12910_2020_454_MOESM1_ESM.docx]

**Additional File**

**Examining the Acceptability and Ethics of**

**Controlled Human Infection Model Research in Malawi**

**Topic guide for focus groups and stakeholder interviews**

**Participants:**

**Interviews:** REC members, hospital managers and clinicians, district health managers

**FGDs:** community advisory groups, frontline MLW researchers, community leaders, local opinion leaders, potential participant groups (medical students).

***'These topic guides were drafted at the outset of the study but used flexibly to respond to issues raised by participants and adapted and refined during the fieldwork in response to emerging findings and reflections on previous interviews'.***

1. **Existing knowledge of CHIM**

- Have you ever heard of CHIM studies?
- If yes, what is it that you have heard about them and know?

1. **Provision of initial information by researchers**

- At this point, researchers with CHIM experience will provide initial background information on CHIM to explain what CHIM research involves.
- Ask for questions and provide answers.

1. **Initial views on CHIM research**

- What do you think about the idea of CHIM research?
- Would conducting CHIM research be OK in Malawi?
- Probe for reasons
  - benefits
  - concerns
  - risks
  - challenges
- Is there anything about the Malawi setting in particular that would affect whether CHIM research is OK?
- Are there any ways that CHIM research could be designed to make it OK in Malawi?
- What would need to be done? How could CHIM studies be designed to be OK?
- Is there anything else that would need to be in place to conduct CHIM studies ethically?
  - Probe on regulatory environment and hospital facilities.

1. **Provision of further information on CHIM and MARVELS plans**

- Ask for further questions and provide information to address any apparent misconceptions to this point e.g. on purpose, risks, benefits, process.
- Explain planned CHIM research in Malawi (MARVELS project) i.e. pneumococcus study starting with healthy volunteers and moving through stages towards vaccine testing with at-risk groups.

1. **Views on specific study plans**

- What are your initial thoughts on these study plans?
- Is there anything you see as problematic?
- Is there anything we particularly need to consider for any of the phases?

1. **Views on specific procedures**

If CHIM studies go ahead, we need to decide how to do this kind of research.

For all the below:

- Probe for reasoning with each question.
- After initial views are given, provide further explanation of the issues and answer any questions, and share planned processes or options being considered to ask whether plans/options would be appropriate for Malawi.
- Inclusion criteria:
- Who could be included in these studies? Are there any people that should not be included? Are there any criteria that mean someone should be included or excluded?
- Probe on education level, age, gender, profession
- Probe on people with HIV
- Recruitment:
- How should information be provided so people hear about the study?
- How should we approach people to ask if they would like to take part in these studies?
- Are there any particular steps that should be followed?
- Consent:
- What would be the most important things for potential participants to understand about CHIM research?
- How could we be sure people understand all the study information?
- Would it be appropriate to test their understanding? If so, how?
- Residential stays:
- Would it be OK to ask participants to stay in a hostel while they are part of the study?
- How long would it be OK for them to stay?
- What would need to be considered to ensure the residential stay is OK?
- Compensation:
- What would be appropriate compensation for CHIM study participants?
- Should this be given all at once at the end, or in stages during their participation?
- Withdrawal: if someone wants to withdraw, there is a risk that they might still be infected.
- How could we handle this? What should be the process if someone wants to withdraw?
- Are there any other aspects of the study we should consider?
- What would the challenge be?
- How could we overcome this?

1. **Community engagement**

- What information should be given to the public about this type of study?
- Are there any issues that would potentially raise concerns?
- Which ones? Why?
- What could be done about these issues?
- Which groups of people should be engaged before a study like this begins?
- What would be the best forums or methods for discussing the study with these people/groups?

1. **Future CHIM**

Beyond the planned MARVELS pneumococcus study, there are options for other kinds of CHIM studies in the future e.g. on other diseases.

- Are there any kinds of diseases that should not be studied using this CHIM method in Malawi? Why – what would be the issues?

1. **Final thoughts**

- Having discussed CHIM and heard more about it, what are your thoughts now on whether this approach would be OK in Malawi?
- Of everything we’ve discussed, which are the most important issues to consider?
- What do you think are the main challenges?
- Is there a way to resolve these challenges?
- Is there anything else you would like to raise?

1. **Closing**

Thank participants for their time

**Topic guide for follow-up interviews**

**Participants:** Follow-up interviews will be conducted with 2-3 participants from each FGD to understand any changes in their views (community advisory groups, frontline MLW researchers, community leaders, local opinion leaders, potential participant groups - medical students).

***'These topic guides were drafted at the outset of the study but used flexibly to respond to issues raised by participants and adapted and refined during the fieldwork in response to emerging findings and reflections on previous interviews'***

1. Have you had any further thoughts on CHIM research since we met for the focus group?
2. What do you think about the idea of CHIM research now?

- Probe on concerns, risks, benefits

1. Is there anything where you changed your mind or where you’ve had new ideas since the focus group?

- What do you think made you shift your ideas?

1. If CHIM studies go ahead in Malawi, what are the most important things to consider when designing these studies?

- Probe on specific procedures – inclusion criteria, recruitment, consent, residential stays, compensation, withdrawal, community engagement.

1. Have you had any discussions about CHIM research with other people since the FGD?

- What impressions did you get about how other people might view this CHIM research?

1. Is there anything else that you suggest we think about or do if we go ahead with planning CHIM studies?
2. Thank participants for their time.
